# Anthropogenic, environmental and temporal associations with vertebrate road mortality in a wildland–urban interface of a biodiverse desert ecoregion

**DOI:** 10.1098/rsos.240439

**Published:** 2024-07-31

**Authors:** Brian R. Blais, Corey J. Shaw, Colin W. Brocka, Samantha L. Johnson, Kayla K. Lauger

**Affiliations:** ^1^ School of Natural Resources and the Environment, University of Arizona, Tucson, AZ, USA; ^2^ Southwest Zoologists’ League, Tucson, AZ, USA

**Keywords:** aridland, development, linear infrastructure, roadkill, urbanization, wildlife conservation

## Abstract

Road mortality adversely affects wildlife populations. As urbanization and infrastructure densities expand, transportation and wildlife management aim to mitigate wildlife–vehicle conflicts while conserving biodiversity. Roadways in aridland ecosystems can invariably and adversely impact wildlife differently from temperate and other biomes, yet these rapidly urbanizing regions are understudied as are urban–rural gradients. We conducted road-cruise surveys (*n* = 204; 2018–2023) to assess anthropogenic, environmental, and temporal factors associated with vertebrate roadkill across the wildland–urban interface of Arizona’s biodiverse Sonoran Desert ecoregion—already subjected to increased human development and climate change. Of *n* = 2019 vertebrates observed, 28.5% were roadkill. Increasing urbanization levels were associated with reduced vertebrate abundance on roads and increased road-killed endothermic vertebrates. Traffic volume was strongly associated with reduced vertebrate abundance and increased roadkill; additive effects on roadkill began at approximately 20 vehicles. Daily low temperature and/or relative humidity were also associated with roadkill across vertebrate groups. We provide empirical evidence to understand wildlife–roadkill associations across expanding wildland–urban interfaces to inform effective roadkill mitigation and wildlife conservation management strategies in biodiverse aridland regions. We recommend that managers mitigate or avoid development in rural areas that possess high biodiversity, valuable waterways or migration corridors, and populations of vulnerable species.

## 1. Introduction

Roads can negatively affect species, communities and ecosystems [1,2], including adjacent vegetation and hydrology [3,4]. Roads affect wildlife by altering habitat (e.g. fragmentation, degradation and loss; [5,6]), or behaviour (e.g. attractance/avoidance of roads; [7–10]) that can lead to intrinsic or extrinsic barriers [11–14]. Road mortality (i.e. roadkill) from wildlife–vehicle collisions is among the greatest threats to wildlife populations [1,15], with greater susceptibility among certain taxonomic forms, demographics (e.g. age- and sex-biases), life history traits (e.g. scavengers, low-flight specialists and nocturnality), or scarcity [6,9,13,16–19]. Herpetofauna (amphibians and reptiles), for example, are particularly threatened by roads [11,20–22], with high mortality occurring during seasonal activity and migration periods [5,23,24].

As urbanization expands and traffic increases, it results in elevated roadkill risks, altered behaviours, and reduced biodiversity across time [10,25]. Assessment of less-developed rural areas and roadways therein is valuable because roadkill risk can remain high owing to speed limits, lower lighting, and abutment to wildlife habitat and natural areas where abundance and diversity is often higher than urban ones [26–28]. The wildland–urban interface describes the transitionary zones between wilderness and open areas to densely populated and developed areas, but receives less attention in road ecology [27,29]. Assessing road ecology in these zones could provide baseline data about wildlife as well as anthropogenic and ecological factors related to road use and roadkill in areas with likelihoods of further development [29,30]. Moreover, road class (e.g. highways versus less-travelled secondary roads) can affect how wildlife responds to roads [16,31,32]. Road ecology studies often focus on single species or single road classes such as highways and high-traffic roads [24,29,31]. Road ecology studies that assess multiple taxa are warranted [33]. Secondary local roads along sparsely developed areas and their effects on wildlife appear to receive less attention. Snakes, which are also under-represented in road ecology studies, for example [20,34,35], are highly impacted by roads regardless of classification or traffic extent [16,22,36].

Environmental factors, such as climate, development, traffic and luminosity, are associated with road mortality [37–41]. Roadkill rates can also vary temporally by diel and seasonal activity periods [42,43]. Many herpetofauna and small mammals, for example, employ nocturnal activity strategies to evade the heat of the day in desert climates [38,44]; herpetofauna often use roads for thermoregulation after the sun sets [34,35,45]. Accounting for temporal and environmental variation is valuable to better understand activity patterns of animals and factors associated with roadkill, which can inform conflict mitigation and conservation strategies [38,46,47].

Much road ecology studies occur in temperate climates, yet roads affect arid and semi-arid regions differently from those in temperate rainy regions [4]. Water accumulation along aridland roads and verges, for example, can facilitate plant growth and microhabitat generation in otherwise open, patchy habitats which can attract wildlife [13]. A greater percentage of taxa are thus at risk of roadkill, especially for species that are attracted to resources along roads (e.g. roadside microhabitat, scavenging opportunities and thermoregulation), move slowly or far for seasonal resources, or are threatened by extinction [13,23,39,48–50]. Diversity in arid regions favours nocturnal species [51], yet, less is generally known about nocturnal ecology of animals [52].

Effective wildlife–vehicle collision mitigation strategies require an understanding of the factors that drive road usage by animals and subsequent roadkill [27,29,33,53,54]. Baseline data in relatively undisturbed areas are useful in comparison with both preserves and urbanized areas [6,35]. To better understand the factors associated with road use and roadkill of aridland wildlife, we conducted temporally replicated post-sunset road-cruise surveys along secondary roads in the wildland–urban interface in the Sonoran Desert ecoregion of Arizona, USA. This aridland system is experiencing both rapid and gradual effects of climate change [55–58], and roads in both urban and rural areas have adversely impacted vertebrate populations [34,35,59–61]. In addition to adding baseline data of road usage by vertebrates along rural and rural–urban edges, our objectives were to (i) compare rates and frequencies of alive to dead detections on roads for four taxonomic groups of terrestrial vertebrates; (ii) model anthropogenic, environmental and temporal factors influencing and leading to vertebrate roadkill; and (iii) assess how anthropogenic development and traffic relate to abundance of observations, which can proxy signals of prior high-mortality events and biodiversity changes [37,62,63]. Understanding such patterns and relationships can provide valuable information for how populations in aridlands respond to expanding development. This work helps to fill multiple gaps in understudied areas of road ecology, including assessment of anthropogenic effects on multiple taxa of various sizes across aridland wildland–urban interfaces [29,31,33,35,59]. Our methodologies can be broadly applied to targeted fauna, ecological communities, and regions, as well as to inform effective wildlife–vehicle collision mitigation and conservation management strategies in aridland ecosystems.

## 2. Material and methods

### 2.1. Study system and sampling design

The semi-arid Sonoran Desert is one of the most biodiverse deserts on Earth [64,65]. The Sonoran Desert ecoregion is characterized by seasonally hot temperatures and bimodal precipitation patterns (i.e. spotty intense summer and prolonged drizzly winter rains), relatively low productivity, and slow plant growth [56,66,67]. Many of its inhabitants employ seasonal strategies (e.g. amphibians and migrating birds) or have adapted their daily activities around climate patterns, such as cathemeral or nocturnal activity periods during the hot summers (e.g. snakes and small mammals; [44,60,68]).

**Figure 1.**
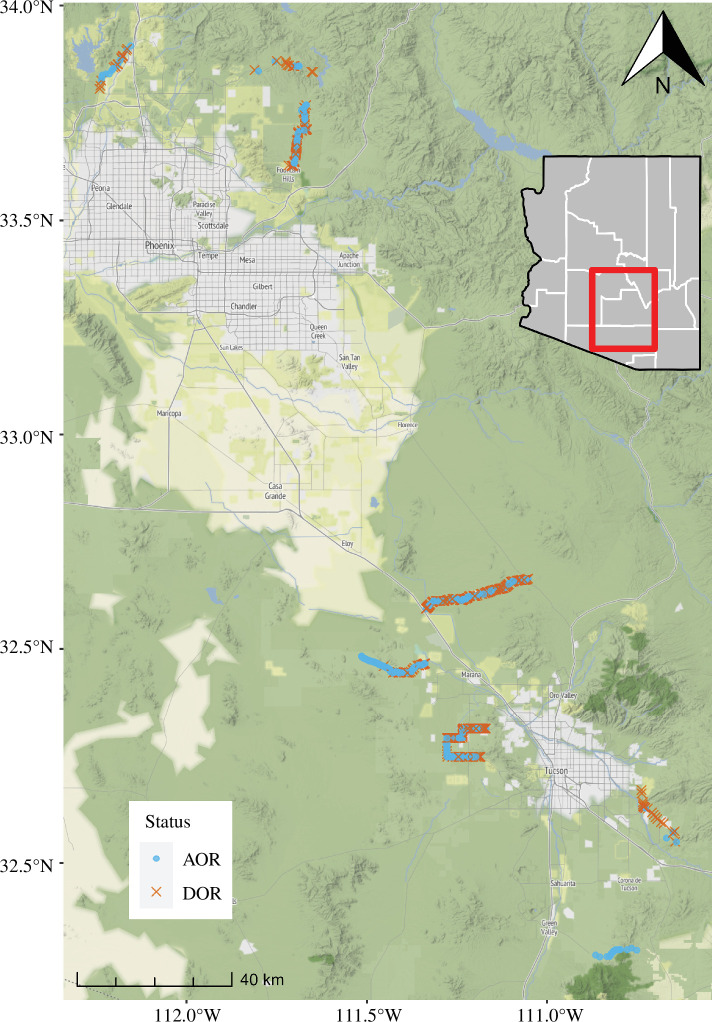
Sampling map of vertebrates detected during evening road-cruise surveys along the wildland–urban interface in the Sonoran Desert ecoregion, Arizona, 2018−2023. Data coloured by detection status, blue for alive-on-road (AOR) and orange for dead-on-road (DOR) vertebrates. Map data © Stamen Design.

We designed a roadway sampling grid comprising eight routes across various extents of the wildland–urban interface of two urban centres in the Sonoran Desert—Phoenix (three routes) and Tucson, Arizona (five routes, less than 1600 m.a.s.l.; figure 1, table 1). The predominant biotic community [69] across routes was Sonoran desertscrub habitat, extending across both Arizona upland (e.g. paloverde-saguaro communities) and lower Colorado River subdivisions (e.g. creosote bush communities; [67,70]); two routes briefly traversed semi-desert grassland and Madrean evergreen woodland communities.

**Table 1.** Road-cruise surveys of vertebrates in the wildland–urban interface of the Sonoran Desert ecoregion, Arizona, 2018−2023. *Route* are the individual transects (*n* = total completed surveys); *urban* is a relative proxy of urbanization level based on residential development and functional road classifications (see the electronic supplementary material); *span* are the years that surveys occurred; *distance* is the transect length, in kilometres; *traffic*, *verts,* and *DOR%* are the mean ± s.d. for vehicles encountered km^−1^ h^−1^; total vertebrates km^−1^ h^−1^; and per-survey percentage of road-killed vertebrates, respectively.

route	urban	span	distance	traffic	verts	DOR%
BD_P_ (*n* = 4)	connecting	2020	21.6	1.59 ± 1.15	0.12 ± 0.22	0.71 ± 0.33
COL_T_ (*n* = 5)	sprawl	2019	22.5	2.80 ± 1.05	0.17 ± 0.22	0.49 ± 0.35
FH_P_ (*n* = 23)	sprawl	2020−2021	20.9	1.80 ± 0.88	0.27 ± 0.17	0.39 ± 0.26
H62_T_ (*n* = 5)	rural	2018	16.6	0.14 ± 0.11	0.20 ± 0.06	0.00 ± 0.00
MW_T_ (*n* = 46)	sprawl	2019−2023	26.3	0.70 ± 0.29	0.30 ± 0.35	0.43 ± 0.23
NR_P_ (*n* = 14)	connecting	2019	16.1	3.14 ± 1.58	0.14 ± 0.13	0.41 ± 0.34
PL_T_ (*n* = 51)	connecting	2018−2023	29.5	0.31 ± 0.18	0.23 ± 0.30	0.35 ± 0.24
SIL_T_ (*n* = 56)	rural	2018−2023	19.3	0.15 ± 0.12	0.37 ± 0.27	0.09 ± 0.12
all (mean ± s.d.)			21.6 ± 4.6	0.80 ± 1.05	0.28 ± 0.28	0.30 ± 0.27

BD, Bartlett Dam; COL, Colossal Cave; FH, Fountain Hills; H62, Highway-62; MW, Mile Wide; PL, Park Link; SIL, Silverbell. Subscripts indicate regional urban centre—P, Phoenix; T, Tucson, Arizona, USA.

Routes consisted of one or more roads, with speed limits less than or equal to 80.5 km h^−1^ (50 mph) and total distances ranging 16.1−29.5 km (total circuit coverage: 172.3 km). We obtained federal functional classifications of roads (i.e. road type) along our routes from Arizona Department of Transportation (ADOT; http://www.azdot.gov). Routes spanned local roads to major collector roads, were no wider than single carriageways (two lanes without meridians), and were either paved, unpaved (graded), or mixed. No roads classified higher than collectors (e.g. arterials and highways) were surveyed; studies among higher functional classifications in the region are described elsewhere [34,59] but see Sullivan *et al.* [35]. Routes transited through relatively undeveloped areas and rural or suburban residential neighbourhoods but were otherwise devoid of businesses or commercial development. All routes were proximal to ecologically important hydrological features (e.g. ephemeral washes, aqueduct canals, artificial reservoirs, and perennial streams) and some ran adjacent to protected natural areas (e.g. Ironwood Forest National Monument, McDowell Mountain Regional Park and Saguaro National Park).

### 2.2. Road-cruise surveys

Between 2018−2023, we conducted systematically replicated vehicle-based surveys (i.e. ‘road cruises’; [34,59]) during the active seasons of many regional fauna, e.g. mid-April to mid-November. We began surveys at least 30 min (mean ± s.d.: 67.1 ± 36.2 min) after local sunset time. This window comprises an optimal period to detect ingressing diurnal, crepuscular, or egressing nocturnal desert fauna [44,65,68] and occurs soon after seasonal roadkill risk times (i.e. evening rush-hour or dusk [71,72]) but probably before peak emergence of nocturnal scavengers [73] (but see Santos *et al.* [74]). We maintained cruising speeds between 32.2 and 48.3 km h^−1^ (20−30 mph; [59]). To reduce spatio-temporal bias, we staggered sampling directions across route surveys. We ceased surveys during moderate to heavy rain. Not all routes were sampled in all years owing to logistical restraints.

Each survey consisted of a single vehicle, led by one of the authors. All vehicle occupants (range: 1−5) scanned the roadways and roadsides until a vertebrate was collectively spotted; i.e. we did not employ independent double-observer sampling. For each detection, we safely stopped to collect data including spatio-temporal data (Universal Transverse Mercator (UTM), World Geodetic System (WGS) 84, accuracy less than 10 m), species and status (alive-on-road (AOR); dead-on-road (DOR)). We identified individuals to taxonomic species when possible but otherwise diagnosed individuals to our most confident level of biological hierarchy if carcasses were not discernable or moving AORs could not be keyed [61].

### 2.3. Anthropogenic, environmental and temporal covariates

For temporal assessment, we designated 15 June−30 September as the wet season corresponding to the North American Monsoon [65], and surveys before or after that timeframe as dry seasons (pre- and post-monsoon, respectively; [58,75]). We considered surveys on Monday–Thursday evenings as weekdays and Friday–Sunday evenings as weekends [41]. We did not survey from December to March owing to inactivity or seasonal absence of local and migrating fauna.

Environmental conditions can influence activity of certain taxa to use roads [41]; the conditions that precede surveys may better represent those experienced by individuals prior to road mortality, especially when time of death is seldom known [74]. We obtained environmental recency data from nearby weather stations for each route (mean station Euclidean distance: 3.9 km). Environmental recency data spanned up to 22 days, corresponding to our mean replication intervals for individual routes. We calculated descriptive statistics for daily temperature (highs and lows), average humidity, and both average and maximum daily precipitation. We also obtained illuminated moon phase (%) corrected for survey time and location from R package *moonlit* [76]. Prior to analyses, we ran a correlation test and principal component analysis (PCA) in the R package *FactoMineR* [77] to determine the collinearity and contribution among the environmental variables, respectively. To mitigate autocorrelation (omittance: *r* ≥ 0.7), we used only mean values for downstream analyses. We retained mean daily low temperature (versus daily high), for example, owing to its representation and contribution in the PCA. Because sparse variation existed in the precipitation data, we applied a binomial condition (yes, no) for rain recency prior to surveys (within 22 days) for downstream analyses.

To estimate traffic, we used hand-counters to quantify the motorized vehicles that passed us in either direction during a survey [78]. This per-survey traffic proxy was strongly correlated (Pearson’s *r* = 0.90) to the average annual daily traffic rates (AADT, obtained from ADOT) along our routes, confirming its utility. To estimate the relative extent of urbanization (i.e. extent of anthropogenic development) along the wildland–urban interface, we categorized route urbanization levels as they related to predominant road type as follows: rural (rural area with little to no residential development alongside roads and of ADOT functional classes no greater than minor collector, i.e. mostly rural local roads); connecting (no residential development but has a major or minor collector road); and sprawl (includes areas with moderate residential development and at least one major collector road). Rural routes averaged less than 250 AADT with speed limits of less than or equal to 56.3 km h^−1^ (35 mph), whereas connecting and sprawl route exceeded 500 AADT with speed limits of less than or equal to 80.5 km h^−1^ (50 mph).

### 2.4. Analyses

Our first objective was to summarize the vertebrate observations and roadkill per taxonomic group, partitioned hereafter as amphibians (e.g. frogs and toads), reptiles (e.g. lizards and snakes) and endothermic vertebrates (electronic supplementary material, table S1). For the latter, we pooled birds (e.g. Caprimulgiformes, Galliformes, Strigiformes and Passeriformes) with mammals (e.g. lagomorphs, mesocarnivores, rodents and even-toed ungulates) owing to insufficient bird data for the finer resolution—a known limitation with avian roadkill [41,47]. The grouping is also logical given bird–mammal nocturnality associations [79], and that most of our avifauna observations comprised cathemeral/nocturnal taxa such as nighthawks, owls, or quail. We used chi-squared test to determine if AOR to DOR ratios were associated among taxonomic groups. Next, we used Kruskal–Wallis and false discovery rate *post hoc* tests to assess equivalent rates of vertebrates among routes, standardized as observations per kilometre per hour. Standardizing rates per unit(s) distance and time have been used elsewhere [61,80], and per hour units may better account for diel and seasonally variable activity patterns of wildlife in our study system [44,61,68].

To understand factors associated with aridland vertebrate roadkill (here, DOR counts per survey) in the wildland–urban interface, we ran generalized additive models (GAMs) with negative binomial distributions in the R package *mgcv* [81]. We used GAMs owing to anticipated nonlinear effects of certain covariates on taxa [41,43,82,83]. Predictors included mean values for environmental recency (i.e. daily low temperature and humidity), precipitation (occurrence), moon fraction (%), season, day of week, urbanization level and total traffic. We included route and year as random effects to account for confounding effects of temporal pseudoreplication [84], and we included route distance as an offset term to account for unequal transect lengths. We omitted observer or the number of observers per survey vehicle in models because preliminary tests revealed no differences in observation rates for those parameters (*p* > 0.05), respectively. We used the restricted maximum likelihood option to account for overdispersion and the gam.check function with 500 replicated simulations to ensure an adequate value for *k* (i.e. maximum degrees of freedom dimensionality) per smoothed covariate [85]. We inspected model diagnostics in the *gratia* package [86]. We compared models using Akaike information criterion (AIC) corrected for GAM use [85]. We used backward selection to remove uninformative priors until models no longer improved [87]. We repeated these modelling steps for individual taxonomic groups.

Because traffic and/or extent of development influences roadkill [27,82], we sought to understand how expanding urbanization levels along aridland wildland–urban interface might also affect road abundance of observed vertebrates (AOR and DOR). Urbanization levels with greater ratios of road-killed observations but fewer overall detections might express signals of past high mortality and elevated extirpation risks [37,62,63]. That is, roadkill rates increase until they negatively affect animal populations [17,21] or behaviours [1,8,32]. Given our classification of rural routes (i.e. relatively undeveloped), we expected connecting and sprawl route levels to exhibit lower total vertebrate rates than rural, thus lending support that urbanization has had disadvantageous effects on vertebrates in those areas. To assess this, we standardized total vertebrates observed per hour and regressed against traffic per hour, urbanization level and their interaction in a generalized linear mixed model in the *glmmTMB* package [88]. To mitigate overdispersion, we used a negative binomial distribution with Broyden–Fletcher–Goldfarb–Shanno (BFGS) optimization and restricted maximum likelihood functions; we included the same random effects and offset as roadkill models. We used the *ggeffects* package [89] to predict counts of vertebrates per hour in relation to urbanization level and traffic per hour from the model results. We used program *R* v. 4.1.1 [90] for all statistical analyses and graphics.

## 3. Results

### 3.1. General findings

Between 2018 and 2023, we completed 204 surveys, covering approximately 4783 km across all routes (table 1). Seasonally, 92 surveys occurred during the wet monsoon season and 112 during seasonally dry periods (pre-monsoon *n* = 71; post-monsoon *n* = 41). Mean survey duration was 1.28 h (s.d. ± 0.44 h) and median observers per survey was 2; total effort exceeded 441 person-hours. During sampling periods, we conducted a survey (any route) every 6 days (s.d. ± 6.55 days); individual routes were replicated every 22.3 days (s.d. ± 16 days). The per-route intervals exceeded known carcass persistence in the area (*ca* 3.2 days; [61]), thus pseudoreplication of individuals was unlikely; most roadkill appeared recent rather than overly desiccated. Overall, we recorded *n* = 2019 individual vertebrate animals comprising 664 amphibians, 340 reptiles and 1015 endothermic vertebrates (figure 1, electronic supplementary material, table S1). Not all routes were equally productive (vertebrates km^−1^ h^−1^: Kruskal–Wallis *H* = 24.3, d.f. = 7, *p* = 0.001; table 1); SIL (Tucson) differed from PL (Tucson; *p* = 0.011) and NR (Phoenix; *p* = 0.011). Vertebrate counts by season were 431, 1418 and 176 for pre-monsoon, monsoon and post-monsoon, respectively.

We recorded 576 DOR vertebrates (28.5% of observations). Ratios of AOR to DOR differed among amphibians, reptiles and endothermic vertebrates (*χ*^2^ = 116.5, d.f. = 2, *p* < 0.001). There were 179 DOR amphibians (27.0% of amphibian observations), 177 DOR reptiles (52.1% of reptile observations), and 220 DOR endothermic vertebrates (21.7% of endotherm observations). Mean roadkill rates (DOR km^−1^ h^−1^: mean ± s.d.) were 0.02 ± 0.06 for amphibians, 0.02 ± 0.04 for reptiles and 0.03 ± 0.04 for endothermic vertebrates; rate for all vertebrates was 0.07 ± 0.09. Urbanization levels also differed in AOR to DOR ratios (*χ*^2^ = 120.1, d.f. = 2, *p* < 0.001); rural routes had a lower DOR ratio than connecting and sprawl routes. We did not partition road substrate type in analyses owing to sample size; unpaved roads made up less than 20% of total sampling effort. We note that of 318 observations on unpaved roads, only eight were DOR (three amphibians, two birds and three reptiles).

### 3.2. Vertebrate roadkill associations

**Figure 2.**
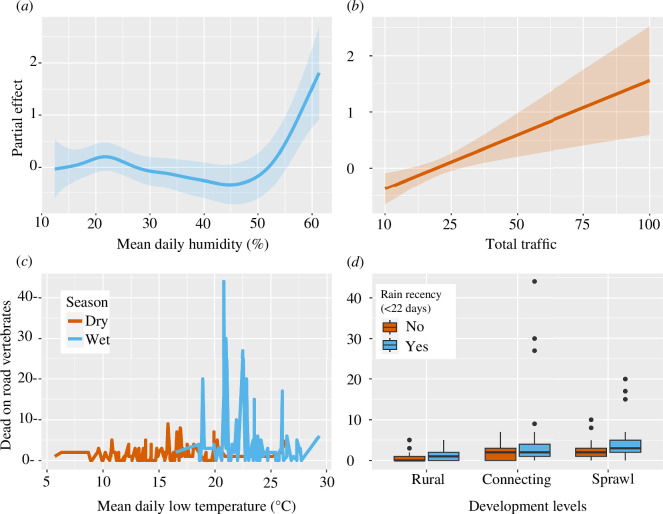
Factors influencing vertebrate roadkill along the wildland–urban interface in the Sonoran Desert ecoregion, Arizona, 2018−2023. Partial effects plots (i.e. isolated effects in additive models; see [81]) for (*a*) mean daily humidity (additive) and (*b*) total traffic (linear) effects on vertebrate roadkill; values greater than 0 indicate a positive effect. Lower panels reflect influences of (*c*) mean daily low temperature seasonality and (*d*) rain on total road-killed vertebrates. Humidity, temperature and rain data were derived from recency data preceding surveys (up to 22 days), i.e. the general environmental conditions leading to fauna use of roads (see the electronic supplementary material).

Roadkill across vertebrate groups in the wildland–urban interface was best explained by a parsimonious model that included traffic, urbanization level, rain recency, and means of daily relative humidity and low temperature as well as their interactions with season, after accounting for random effects and offset term (48.2% of variance; electronic supplementary material, table S2). There was a nonlinear increase in vertebrate roadkill when mean daily humidity exceeded 52.4% after accounting for other covariates (*p* = 0.002; figure 2*a*, table 2). Vertebrate roadkill increased linearly with traffic, with positive effects occurring with at least 20 cars/survey (*p* = 0.003; figure 2*b*). Mean traffic per hour during this project was 17.2 (s.d. ± 19.5); we estimated that for every 100 vehicles, there would be 6.1 (95% confidence interval (CI): 2.1−17.9) road-killed vertebrates across our study system. Mean daily low-temperature recency was not a strong predictor of vertebrate roadkill (*p* = 0.236) except when there was a seasonal effect (*p* = 0.045; figure 2*c*). The occurrence of rain preceding a survey (within 22 days) increased the odds of roadkill counts by 1.42 times (s.e. ± 1.18) than without recent rain (figure 2*d*). Rain recency (*p* = 0.016) was a better predictor than seasonal effects alone (*p* = 0.098; table 2). Despite differing AOR to DOR ratios, increasing extent of urbanization level was non-significant in the DOR model when considering all vertebrate groups (*p* > 0.05, table 2).

**Table 2.** Covariate metrics in best-performing GAMs of vertebrate roadkill in the wildland–urban interface of the Sonoran Desert ecoregion, Arizona, 2018−2023; all models account for random effects of year and route as well as an offset (route distance). Taxonomic groups include A = amphibians, E = endothermic vertebrates (birds, mammals), R = reptiles and V = all vertebrates. Values are converted log odds for parametric factors and estimated degrees of freedom (edf) for smoothed and interaction terms; “—” = covariate unused in optimal model; *p*-values are shown in parentheses. Covariates are described in the electronic supplementary material.

type	covariate	A	R	E	V
factors (parametric)	urbanization (connecting)	0.75 (0.65)	—	2.36 (0.02)	1.78 (0.42)
	urbanization (sprawl)	2.95 (0.11)	—	3.57 (<0.01)	2.41 (0.24)
	rain^a^ (yes)	9.32 (<0.01)	—	1.40 (0.06)	1.50 (0.02)
	season (wet)	18.97 (<0.01)	—	—	1.61 (0.10)
	week (weekend)	—	—	—	—
smooths	total traffic	1.00 (0.08)	2.07 (0.05)	—	1.00 (<0.01)
	humidity^a^	2.70 (0.04)	2.67 (0.12)	2.91 (0.05)	4.85 (<0.01)
	moon phase	—	—	—	—
	temperature^a^	—	4.76 (0.01)	1.00 (<0.01)	1.00 (0.24)
tensor interactions	humidity^a^ × season	—	2.82^−5^ (0.02)	—	5.28^−6^ (0.77)
	temperature^a^ × season	—	5.32^−5^ (0.48)	—	1.22 (0.05)

^a^ Derived from condition averages during and preceding surveys (recency up to 22 days, i.e. mean interval between individual route replication), comprising daily mean humidity, daily low temperature, and whether rain occurred or not (binomial).

Among taxonomic subsets, roadkill of endothermic vertebrates (birds and mammals) was best explained by a parsimonious model with urbanization level, rain recency, and mean daily low temperature and humidity (27.4% variation; electronic supplementary material, table S2). Three competing models were within 2 AIC units, but their additional parameters failed to improve models or variance explained (electronic supplementary material, table S2). Compared with rural routes, roadkill of endotherms was 2.36 (s.e. ± 1.45) times more likely in connecting urbanization levels (*p* = 0.022) and 3.57 (s.e. ± 1.44) times more likely in sprawl areas (*p* < 0.001; table 2). The odds of endothermic roadkill trended increasingly with recent rain by 1.40 (s.e. ± 1.20) times than without rain (*p* = 0.060). Relative humidity had a nonlinear effect on endotherm roadkill—increasing when below 26% and above 52% (*p* = 0.048; electronic supplementary material, figure S1*a*). Daily low temperature had a significant linear effect on endotherm roadkill, specifically at or above 18.6°C (*p* = 0.002; electronic supplementary material, figure S2*a*; table 2).

Amphibian roadkill was highly influenced by seasonal effects. The top model for amphibians explained 69.1% of variation in the data and included the covariates: season, rain recency, mean daily humidity, urbanization level and traffic (electronic supplementary material, table S2). Competing models with additional parameters failed to improve results. After accounting for additive effects, amphibian roadkill was 19.0 (s.e. ± 1.7) times more likely to occur during the wet monsoon season than the dry seasons that flank it and 9.3 (s.e. ± 1.9) times more likely when rain occurred prior to surveys (table 2). Amphibian roadkill also increased with mean daily humidity (greater than or equal to 43%, *p* = 0.036; electronic supplementary material, figure S1*b*). Road mortalities trended with increased hourly traffic (*p* < 0.084; electronic supplementary material, figure S2*b*).

Two competing models best explained reptile roadkill (28.4−31.1% variance) and included traffic, mean daily relative humidity and low temperature, and their interactions with season; the addition of urbanization and season factors in the competing model failed to improve results (electronic supplementary material, table S2). Reptile roadkill increased with mean daily low temperature, having additive effects for temperatures between 15°C and 23°C and especially above 27°C (*p* = 0.015; electronic supplementary material, figure S2c; table 2). Reptile roadkill trended increasingly with mean daily humidity above 22% (*p* = 0.119; electronic supplementary material, figure S1*c*), and significantly for seasonal interaction with humidity (i.e. during the rainy season; *p* = 0.023). Traffic also influenced reptile roadkill (*p* = 0.049); an effect was detected at greater than or equal to 15 cars (electronic supplementary material, figure S2*d*).

### 3.3. Effects of traffic and urbanization on vertebrate occurrence

**Figure 3.**
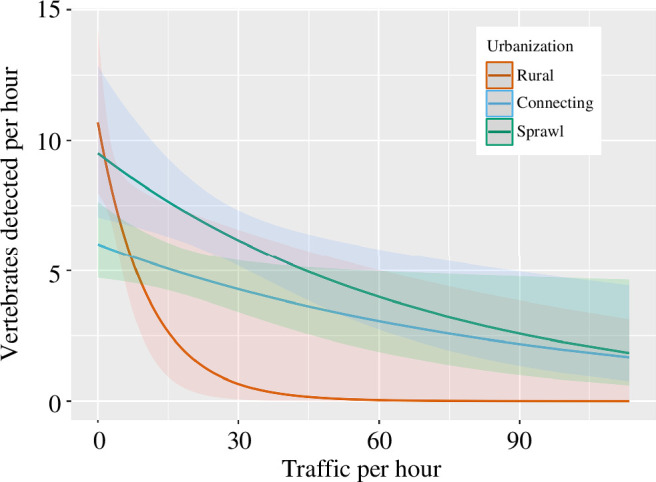
Predicted effects of traffic on the number of vertebrate observations per hour on roads in the wildland–urban interface of the Sonoran Desert ecoregion, Arizona. Coloured lines and shaded regions depict the prediction and CIs per urbanization level, respectively (see the electronic supplementary material).

The number of vertebrates detected per hour was related to traffic per hour and urbanization level, after controlling for random effects of route and year (*R*^2^*c* = 0.335). For each unit increase in hourly traffic, vertebrates per hour declined by 10.2% (95% CI: 2.1−17.7%, *p* = 0.014; electronic supplementary material, figure S3). For routes in connecting urbanization levels, the vertebrate rate was 0.457 times as much as in rural levels—i.e. 54.3% (95% CI: 31.6−69.4%) fewer vertebrates per hour—while holding other variables constant (*p* < 0.001; figure 3). Vertebrates per hour in sprawl areas trended 0.711 (−28.9%) times as much as rural, but with some uncertainty (i.e. from −54.6% decline to 11.3% increase; *p* = 0.136). Hourly traffic rates were greater in connecting (mean ± s.d. = 19.0 ± 21.6, *p* = 0.025) and sprawl (27.4 ± 18.4, *p* = 0.038) urbanization levels than rural (2.9 ± 2.3), respectively. For perspective on traffic impact across our study, predicted vertebrates per hour (any status) was 7.7 (95% CI: 5.4−11.0) at one vehicle per hour, declining to 3.4 (95% CI: 1.8−6.1) vertebrates at 20 vehicles per hour. Less than one vertebrate per hour was predicted at greater than or equal to 48 vehicles per hour.

## 4. Discussion

Assessing the factors linked to roadkill can better inform effective conservation management strategies [1,6,33,54], especially at multi-species scales and in understudied systems, such as aridlands [2,4,13,40]. Here, we explored anthropogenic, environmental and temporal scale variables associated with roadkill of vertebrates in an aridland wildland–urban interface already subject to expanding human development and climate change [30,91]. We found that increased levels of urbanization were associated with road-killed endotherms but not ectotherms and in part with abundance of vertebrates observed on roads. Rather, traffic was more strongly associated with reduced vertebrate abundance and increased vertebrate roadkill. Daily low temperature and/or relative humidity were also linked to roadkill of endothermic or exothermic vertebrate groups to various degrees. The empirical evidence herein can be used in guiding transportation and wildlife conservation management strategies to understand and mitigate roadkill in the biodiverse wildland–urban interface in aridland systems like the Sonoran Desert.

### 4.1. Environmental influences on vertebrate roadkill

Environmental factors can help predict vertebrate roadkill across the wildland–urban interface in Arizona’s Sonoran Desert ecoregion. It is difficult to know exactly when an animal is struck or killed on roadways, and weather data collected at time of roadkill detection may not reflect the conditions experienced prior to death [36]. By assessing weather trends preceding detection, we better account for the environmental parameters that led to animals using a roadway [39–41]. In this study, relative humidity and ambient temperature metrics were among the top environmental predictors of roadkill; these variables are known to influence activity of ectothermic [37,38,92] and endothermic vertebrates [44,51,93]. Humidity was a strong predictor for amphibians, for example, as this relationship corresponds to known life history and phenology patterns [38,53]. Roadkill of endothermic vertebrates was greatest at lowest and highest levels of humidity but not at intermediate ranges. This may be owing to the drastic seasonal changes in a region affected by a wet summer monsoon season flanked by dry spring and autumn seasons [91]. Elsewhere, road-killed birds have been negatively linked to increased humidity and mammal roadkill is positively linked to humidity/precipitation and temperature [38,40,46]. Further deciphering the types of humidity, such as those in microclimates experienced by mammals [44], may resolve the nuances of such relationships.

Temperature may better explain mammalian activity, which diminishes towards the extremes [44,51]. Here, roadkill of endothermic vertebrates as well as reptiles was positively influenced by daily low temperature—a trend observed in these classes elsewhere [38,40,42,92]. Our post-sunset sampling times coincided with when many Sonoran Desert reptiles, especially snakes, increase activity owing to more tolerable temperatures [60,65,68] and nocturnal mammals begin their surface activities [44,94]. Crepuscular and nocturnal taxa thus move onto roads for thermoregulation, transit, or by chance [31,35,59]. Of note, moon phase did not influence roadkill of any vertebrate group, but we did not assess AOR or species-specific relationships, which could uncover avoidance or ignorance behaviour patterns related to luminosity [95–97]. We also acknowledge that environmental conditions can influence both scavenger activity and carcass degradation times [74,98], which could shortfall the reality of roadkill in this system.

Numerous species have shown behaviour shifts in relation to changes in atmospheric conditions [99,100]. Species whose activities are influenced by seasonal climate patterns, such as those related to the North American monsoon, may face greater susceptibility to roadkill. Increases in humidity and precipitation during the summer monsoon triggers activity—and road use—by herpetofauna in the Sonoran Desert, especially amphibians [35,59,68]. Many desert amphibians breed in ephemeral pools created during summer rains, followed by dispersal thereafter [101,102]. Amphibian roadkill was more probable after periods of rain in this study, suggesting that the risks of mortality for amphibians are sustained near seasonal aquatic habitats. Increased reptile roadkill was positively linked to humidity during the wet summer season but not during the drier seasons that flank it. The effects of seasonality on certain taxa lends importance to longitudinal studies [30,38].

### 4.2. Anthropogenic urbanization effects across a wildland–urban interface

Understanding how anthropogenic development and urbanization affects wildlife populations is important for effective conflict mitigation and conservation strategies. Increasing development and linear infrastructure brings about many conditions that can affect wildlife, including noise, lighting and resource sinks, and is often synonymous with decreasing biodiversity [1,2,5,30]. Urbanization can, in turn, facilitate demographic strains that may lead to population declines and extirpation, ultimately resulting in diversity changes in favour of ‘urban adapter’ species [10,103]. Arid environments are among the swiftest to be urbanized, yet among the most vulnerable to climate change and natural disasters [94,104]. In the wildland–urban interface of the Sonoran Desert, our prediction that increased levels of urbanization would result in fewer total vertebrate observations was partly supported. Although routes in connecting urbanization levels yielded significantly less abundance of vertebrate observations than rural routes, sprawl levels did not, albeit with descending abundance trends. However, this finding does not account for the potential diversity shifts between wildland areas versus those more developed or towards urban adapted or less-sensitive species; species-specific trends was not the focus of this study. If wildlife communities have shifted across the Sonoran Desert’s wildland–urban interface, it may indicate that urbanization and past roadkill events have had detrimental impacts to biodiversity [37,62,63]. Endotherm roadkill was positively linked to increasing urbanization extent but not traffic.

Traffic appears to have strong effects on fauna in the Sonoran Desert ecoregion. Traffic rates significantly increased with urbanization levels. That is, more urbanized development levels equated to greater volumes of traffic, and busier roads resulted in fewer detections of vertebrates of any status. Traffic volumes can affect species differently based on their behaviour pertaining to traffic associations, usually resulting in direct mortality or increased barrier effects [105].

Traffic is a leading factor of roadkill of numerous taxa across many regions and habitats [7,41,106–108], including the Sonoran Desert ecoregion [34,59,61]. As road densities increase and further bisect wildlife populations and corridors, roadkill rates increase [27,33,109]. Despite the relatively low traffic volumes on surveyed routes, traffic was a strong predictor of vertebrate roadkill in this study. Increased effects began at approximately 20 vehicles per survey, i.e. approximately 1.3 hours and 21.6 km on average. Weekends versus weekdays did not influence roadkill, suggesting equivalent risks on any given day of the week.

Herpetofauna were especially vulnerable to traffic. Reptiles—predominantly snakes but also lizards—had the highest frequencies of DOR. This is not surprising given that reptiles are commonly found inactive on roads (e.g. thermoregulation) or traverse them slowly, all of which probably increases risk of road mortality [33,107,110]. Herpetofauna sometimes move just a short distance onto a roadway for thermoregulation or other resources and become immobile (i.e. inactive; [45,111])—unfortunately, this often aligns with the travel pathway of vehicle tyres. Though, snakes and some other taxa are struck by vehicles owing to drivers intentionally swerving outside of the transit path [78,112]. We found many amphibians inactive on roads, yet their DOR ratios were much lower than reptiles. We attribute this to their prolific fecundity-driving abundance and small stature, i.e. amphibians appear less likely to encounter vehicle tyres than long, tubular snakes. Even in locations of high amphibian mortality, such as adjacency to ephemeral breeding pools during episodic movements following metamorphosis events, many unharmed individuals remained on or alongside roadways. However, risks of road mortality for aridland amphibians are likely sustained as wetland adjacency and low traffic roads can yield high roadkill rates in herpetofauna [36,107]. Some animals (e.g. ravens, some mesocarnivores, and rodents) tend to avoid roads, cross them more swiftly, or have behavioural responses to oncoming traffic, i.e. they move out of the way [7,31,33,105]. Escape response, however, may not always match oncoming vehicle speed [113,114]. Assessing placement in roadways by inactive or DOR animals would be informative to further understand cues and causes for roadkill risk.

### 4.3. Conservation implications and future concerns

As urbanization expands across the wildland–urban interface and road densities subsequently increase [115], transportation and wildlife managers must balance biodiversity conservation while mitigating wildlife–vehicle collisions [6,27]. An understanding of when, where and why wildlife use roads can guide mitigation planning for management activities, identify spatio-temporal patterns and inform conservation strategies [29,33,53,54,116]. Wildlife–vehicle collision mitigation and road effect reduction strategies are discussed elsewhere [2,54,117], but we note that some roadkill mitigation measures (e.g. overpasses) have been initially effective in the Sonoran Desert ecoregion [117,118]. Here, we focused on anthropogenic, environmental and temporal factors associated with roadkill across biodiverse vertebrate groups in an aridland wildland–urban interface. Although species-specific associations with roadkill may provide further resolution of its intricacies, we focused at the class scale to derive a baseline understanding of roadkill effects on biodiversity in an aridland ecosystem [40]. Spatio-temporal assessment of road ecology for individual taxa was not a focus of this article, but studies are ongoing (BR Blais, unpublished data).

Environmentally, a significant conservation concern for biodiversity revolves around ongoing climate change and the stressors it brings upon wildlife. Climate in many arid regions, such as the American Southwest, is predicted to become hotter and drier and with fluctuations towards extreme events [56,119–122]. Increasing temperature and aridity can alter soil pH [66], plant phenology and productivity [123,124], and community compositions [30,125]. Small mammals may be better equipped for climate change than birds, for example, owing to their abilities to use microhabitat refuges to escape heat [93]. Increasing temperature, especially near physiological limits, however, could bring unavoidable heat stress and disadvantageous health effects to small mammals, which can reduce the time needed to exercise at tolerable temperature [44,94]. Here, increasing temperature was associated with greater roadkill for endothermic vertebrates and reptiles.

Climate change has already affected numerous species [93,126,127], and species must move, adapt, or collapse [128,129]. The Sonoran Desert ecoregion has experienced several extreme disturbances in recent years [58,130], and fauna are experiencing increased exposure to high temperature in urban settings or wildlands [91,94]. As days become hotter and drier, prolonging or exceeding the conditions at upper thermal maxima of species, could it shorten the activity windows of cathemeral or nocturnal species or cause diurnal taxa to shift activity further away from daytime extremes? During this project, we observed multiple desert iguana (*Dipsosaurus dorsalis*)—a predominantly diurnal, heat-tolerant lizard—inactive on roads after dark [75]. If species indeed shift or compact their activities to more tolerable diel periods, and thus encounter roads by chance or intentionally during higher traffic periods (e.g. commuter rush-hour), roadkill risks may increase, especially for taxa already vulnerable to roads.

Assessing the extent of anthropogenic development and various road classifications on road use and roadkill of animals facilitates our understanding of the subtle changes in biodiversity across an urban–rural gradient. This is important in areas affected by climate change, where increasing urbanization can necessitate greater demands on natural resources [55,57,58]. Anthropogenic development and climate may already be affecting arthropod communities at urban edges in the Sonoran Desert [30]. Our urbanization design, including road classification and traffic rates therein, could be viewed in a spatio-temporal sense—that is, as ‘before and after impact’ proxies of development [131]. Rural designations in this study best represented the wildland edges of the wildland–urban interface owing to sparse residential development and limited through-traffic. These routes and areas can thus be viewed as the ‘before’ expanded development. For example, the rural SIL route bypassed an important ephemeral wash on the way through Ironwood Forest National Monument and yielded some of the lowest traffic and DOR ratios with the highest abundance of vertebrate observations. In contrast, other routes in more developed areas—those with higher functional road classes and/or moderate residential development—are probably representative of ‘after-effects’ of urbanization and habitat encroachment. These areas may be newly urbanized and still yield high abundance but high road mortality or have had enough urban sprawl effects where abundances and communities have shifted, either by behaviour and indirect effects of road infrastructure or signals of past high mortality [37,62,63]. Assessing urbanization level alone may not elucidate the relationships between biodiversity and threats therein. Other factors are necessary to make more informed decisions.

A concerning finding in this study was how little traffic it takes to generate adverse returns on detection abundance or roadkill incidences. We detected twofold negative effects at only 20 vehicles per survey—one being a reduction of vertebrate observations of any status and two, an increase in DOR incidents. Both connecting and sprawl urbanization levels exceeded this traffic rate per survey, and the effects on abundance and mortality of vertebrates therein was evident. Because carcasses can be missed or are removed more frequently than our survey intervals [26,28,61], we acknowledge imperfect detection [63] and our results probably underestimate the number of vertebrate roadkill. Rather, we focused on modelling associations with observed roadkill and not to project estimations of annual roadkill tallies, population demographics, or carcass disposition (but see Gerow *et al.* [61]).

In the wildland–urban interface of Arizona’s Sonoran Desert ecoregion, adverse effects of roads on vertebrates are likely to be exacerbated by ongoing human population growth, expanding development, and encroachment into natural habitats [61,70,132,133]. This includes known impacts to wildlife populations around the existing Interstate−10 corridor [59,60,134,135] as well as likely effects from the proposed I−11 through Arizona [133,136]. The I−11 developers’ preferred pathway option (http://i11study.com/Arizona/index.asp) would directly splice or come in close proximity to some of our survey areas and important ephemeral stream reaches where we detected many amphibians during their breeding season, such as *Incilius alvarius*, a species of greatest conservation need [68,110]. The preferred I−11 option would also bisect several biodiverse and ecologically important areas in Arizona (e.g. Hassayampa ecosystems, Ironwood National Forest, Santa Cruz River valley, Sonoran Desert National Monument, and Tohono O’odham Nation; [137,138]) and complete an encirclement of high-volume roadways around Saguaro National Park–West District. The latter is home to several threatened taxa, and an estimated 30 000 vertebrates are killed annually on rural roads just in the vicinity of this National Park alone [61]. Given the findings that increased traffic is highly detrimental to wildlife, especially herpetofauna, along major roadways [34,59] and less-travelled secondary roads (this study) in the Sonoran Desert, the preferred I−11 option would undoubtedly and unfavourably lead to increased roadkill and threaten to alter the area’s biodiversity.

Planning of new linear infrastructure in aridland ecosystems should exercise caution around roadway location, especially near hydrological networks. Roads that interrupt hydrological sheet-flow and collect water can attract wildlife [3]. In turn, this can increase roadkill risks, especially where roads bisect important environmental areas such as sources of water, protected areas, presence of threatened species, and movement corridors [4,13]. Waterways and riparian zones in semi-arid Arizona are hotspots for diversity [139]; many of our amphibian observations occurred near perennial or seasonal streams and wetlands. Increased traffic was strongly associated with reduced abundance of observed vertebrates and increased roadkill across the wildland–urban interface of southern Arizona. Transportation planners and wildlife managers should consider how new or expanding linear infrastructure projects and subsequent traffic may affect local biodiversity in aridland systems, especially around protected areas [140]. We recommend that transportation managers mitigate or avoid developing or expanding linear infrastructure in rural areas that possess high biodiversity (e.g. nature preserves), valuable permanent or seasonal waterways that serve as breeding grounds or migration corridors, and populations of imperilled species or those with high roadkill risks.

## Data Availability

The datasets and code supporting this article are available in the supplementary material [141].
